# Apoptotic Vesicles Derived from Dental Pulp Stem Cells Promote Bone Formation through the ERK1/2 Signaling Pathway

**DOI:** 10.3390/biomedicines12040730

**Published:** 2024-03-25

**Authors:** Kunkun Yang, Yuan Zhu, Yuzi Shao, Yuhe Jiang, Lei Zhu, Yaoshan Liu, Ping Zhang, Yunsong Liu, Xiao Zhang, Yongsheng Zhou

**Affiliations:** 1 Department of Prosthodontics, Peking University School and Hospital of Stomatology, No. 22, Zhongguancun South Avenue, Haidian District, Beijing 100081, China; yangkunkun@pku.edu.cn (K.Y.); zhuyuan@pku.edu.cn (Y.Z.); shaoyuzi@hsc.pku.edu.cn (Y.S.); jiangyuhe@pku.edu.cn (Y.J.); 18800183672@163.com (L.Z.); liuyaoshan@stu.pku.edu.cn (Y.L.); zhangping332@bjmu.edu.cn (P.Z.); liuyunsong@hsc.pku.edu.cn (Y.L.); 2 National Center of Stomatology, National Clinical Research Center for Oral Disease, National Engineering Research Center of Oral Biomaterials and Digital Medical Devices, Beijing Key Laboratory of Digital Stomatology, NHC Key Laboratory of Digital Stomatology, NMPA Key Laboratory for Dental Materials, No. 22, Zhongguancun South Avenue, Haidian District, Beijing 100081, China

**Keywords:** dental pulp stem cells, apoptotic vesicles, osteoporosis, bone defect, osteogenic differentiation, ERK1/2 signaling pathway

## Abstract

Osteoporosis is a common degenerative bone disease. The treatment of osteoporosis remains a clinical challenge in light of the increasing aging population. Human dental pulp stem cells (DPSCs), a type of mesenchymal stem cells (MSCs), are easy to obtain and have a high proliferation ability, playing an important role in the treatment of osteoporosis. However, MSCs undergo apoptosis within a short time when used in vivo; therefore, apoptotic vesicles (apoVs) have attracted increasing attention. Currently, the osteogenic effect of DPSC-derived apoVs is unknown; therefore, this study aimed to determine the role of DPSC-derived apoVs and their potential mechanisms in bone regeneration. We found that MSCs could take up DPSC-derived apoVs, which then promoted MSC osteogenesis in vitro. Moreover, apoVs could increase the trabecular bone count and bone mineral density in the mouse osteoporosis model and could promote bone formation in rat cranial defects in vivo. Mechanistically, apoVs promoted MSC osteogenesis by activating the extracellular regulated kinase (ERK)1/2 signaling pathway. Consequently, we propose a novel therapy comprising DPSC-derived apoVs, representing a promising approach to treat bone loss and bone defects.

## 1. Introduction

In recent years, the number of patients with bone loss and bone defects among the aging population has markedly increased, seriously affecting their health and quality of life. Osteoblasts and osteoclasts are primarily responsible for the growth, development, and metabolism of bone. Through intricate interactions, they enable the body to create new bone tissue and absorb old bone tissue in a dynamic balance [1,2]. Osteoblasts are differentiated from mesenchymal stem cells (MSCs), which secrete specific active substances, such as calcium phosphate, mineralize new bone, and promote bone regeneration [3]. 

Several signaling pathways coordinate to cause the osteogenic differentiation of MSCs, such as the Janus Kinase (JAK)/signal transducer and activator of the transcription (STAT) signaling pathway, the phosphatidylinositol-3-kinases (PI3K)/protein kinase B (AKT) signaling pathway, and the extracellular regulated kinase (ERK)/mitogen-activated protein kinase (MAPK) signaling pathway [4,5,6]. Exosomes derived from human alveolar bone-derived bone marrow mesenchymal stromal cells (AB-BMSCs) activate the PI3K/AKT signaling pathway, which stimulates the osteogenic differentiation of bone marrow mesenchymal stem cells (BMMSCs) and promotes new bone formation [7]. Furthermore, a study found that the deletion of the *Stat3* gene in pre-osteoblasts inhibited their differentiation into osteoblasts, and then hindered bone formation through the JAK/STAT signaling pathway [8]. BMMSC-EVs inhibited the ERK1/2 signaling pathway by delivering miR-206 into osteosarcoma cells, thus inhibiting the progression of osteosarcoma [9]. A range of growth factors, anti-inflammatory factors, and anti-catabolic factors can be produced by MSCs through a paracrine response to the signaling pathways involved in osteogenesis.

However, currently, the application of MSCs faces significant preclinical and clinical hurdles, such as the trapping of cells in non-target organs, low survival rates, and uncertainty over MSCs’ fate following transplantation [10,11]. After being administered in vivo, MSCs have the potential to undergo apoptosis and release apoptotic vesicles (apoVs) which can help alleviate diseases [12,13]. ApoVs are extracellular vesicles (EVs) produced by apoptotic cells, and possess a lipid bilayer membrane containing biological components such as mRNAs, DNAs, lipids, and proteins, which play important roles in signal transduction and homeostatic regulation [14,15,16]. 

Human dental pulp stem cells (DPSCs) are a type of MSCs that can be extracted from the third molar tooth, primary teeth, and supernumerary teeth, with little harm to the donor, and can differentiate along the usual mesodermal cell lineage [17]. Compared with classic BMMSCs DPSCs exhibit a higher proliferation rate [18]. In addition, DPSC-derived EVs were reported to show stronger immunoregulatory effects than BMMSC-derived EVs [19]. Therefore, DPSC-derived EVs might be acquired more conveniently, with higher a production rate and increased bioactivities compared to BMMSC-derived EVs. DPSC-derived EVs have also been documented to exert therapeutic effects on skin wounds, spinal cord injury, and mandible and calvarial bone defects [20,21,22,23]. DPSC-derived EVs induced by hypoxia showed good potential to treat inflammatory or infectious osteolysis, and the study determined a new role for miR-210-3p in inhibiting the expression of nuclear factor kappa B1 (NF-κB1) while inhibiting osteoclast generation and the inflammatory response of macrophages [24]. DPSC-derived exosomes can polarize macrophages from the M1 phenotype to the M2 phenotype, which could effectively reduce alveolar bone loss and promote periodontal epithelial healing in experimental periodontitis rats [25]. 

Compared with exosomes, apoVs have a high yield, an abundant quantity, and are easy to extract. Previously, we established a unique protein map of apoVs from MSCs and determined the differences between apoVs and exosomes in terms of functional cargo proteins and surface markers [12]. Our group found that BMMSC-derived apoVs released miR1324 and activated the SMAD family member 1/5 (SMAD1/5) pathway in target cells to induce bone regeneration. BMMSC-derived apoVs can alleviate bone loss caused by primary and secondary osteoporosis and stimulate bone regeneration in bone defect areas [26]. Recently, it was found that DPSC-derived apoVs can activate recipient cells, thus inducing host angiogenesis and promoting pulp regeneration in hypoxic–ischemic environments [27]. However, the ability of DPSC-derived apoVs to stimulate the osteogenic differentiation of MSCs and enhance bone repair via certain osteogenic differentiation signal pathways in osteoporosis disorders is mostly unclear.

The transplantation of MSCs can generate exogenous apoVs capable of regulating endogenous MSC function and maintaining bone homeostasis [15]. However, the detailed functional role of DPSC-derived apoVs is largely unknown. Therefore, we investigated the regulatory role of DPSC-derived apoVs in bone metabolism and their therapeutic effects on bone defects and bone loss. Our results provide an important theoretical basis for novel clinical applications of DPSC-derived apoVs.

## 2. Materials and Methods

### 2.1. Cell Culture

ScienCell Research Laboratories (Carlsbad, CA, USA) provided the human BMMSCs and human DPSCs used in our investigation, and all the in vitro experiments were performed three times. Sigma-Aldrich (St. Louis, MO, USA) provided all the components for cell culture. Fetal bovine serum (FBS; 10%, vol/vol), penicillin G (100 U/mL), and streptomycin (100 mg/mL) (all Gibco, Grand Island, NY, USA) were added to Minimal Essential Medium to form the proliferation medium (PM). After reaching 80–90% confluence, the cells were cultured in osteogenic medium (OM), comprising PM supplemented with 0.2 mM ascorbic acid and 10 mM glycerophosphate. The conditions for cell culture were 5% CO_2_, 95% air, 37 °C, and 100% relative humidity.

### 2.2. Isolation and Purification of apoVs Derived from DPSCs

We purchased staurosporine (STS) from Enzo Life Sciences (Farmingdale, NY, USA). The addition of STS for 12 h caused apoptosis in DPSCs that had attained 100% confluence. According to an earlier investigation, apoVs were recovered by differential centrifugation from the supernatants of DPSCs [12]. Cells and cellular debris were removed from the supernatant by centrifuging it at 800× *g* and 2000× *g* for 10 min. The supernatant was subjected to a 30 min ultracentrifugation at 16,000× *g* and the pellet was washed using phosphate-buffered saline (PBS), followed by centrifugation at 16,000× *g* [12,15]. The concentration of apoVs derived from DPSCs was determined using a Pierce BCA Protein Assay Kit (Thermo Fisher Scientific, Waltham, MA, USA).

### 2.3. TdT-Mediated dUTP Nick End Labeling (TUNEL) Staining

DPSCs were seeded on the sterile glass coverslips placed on 24-well plates. The samples were cultured with STS for 2 h before being rinsed three times in PBS, fixed in 4% paraformaldehyde at 4 °C for 30 min, and then permeabilized in 0.1% Triton X-100 for 15 min. The samples were then treated with TUNEL dye (Applygen Technology, Beijing, China). The nuclei were labeled with 4′,6-diamidino-2-phenylindole (DAPI; Zhong Shan-Golden Bridge Biological Technology, Beijing, China), and the coverslips were placed on glass slides. Images were acquired using a Zeiss Axiovert 650 confocal microscope (Carl Zeiss, Oberkochen, Germany).

### 2.4. DPSC-Derived apoVs Uptake by MSCs In Vitro 

ApoVs were labeled with PKH-26 (Sigma-Aldrich), a red fluorescent cell linker. In accordance with the manufacturer’s instructions, apoVs and 4 μL of PKH-26 dye were incubated together in 1 mL of Diluent C. To bind excess dye, the dilutions were gently mixed for 4 min before adding 2 mL of 0.5% bovine serum albumin (BSA). The labeled apoVs were then washed in PBS at 16,000× *g* for 30 min. Following that, the apoV pellet was incubated with MSCs for 4, 8, and 12 h, respectively. After incubation, the cells were washed twice with PBS and then fixed in 4% paraformaldehyde for 10 min before being rinsed again. The cell nuclei were stained with a 1 g/mL solution of DAPI. Images were acquired using the Zeiss Axiovert 650 confocal microscope.

### 2.5. Identification of DPSC-Derived apoVs

Transmission electron microscopy (TEM) was used to investigate the morphology of apoVs. DPSC-derived apoVs were dropped on copper grids coated with carbon after being treated with 2% paraformaldehyde for 30 min. After air-drying, the mixture was subjected to two applications of 1% uranyl acetate for negative staining. A Hitachi HT7700 TEM (Hitachi, Tokyo, Japan) was used to acquire images at 120 kV. Using the ZetaView system (Particle Metrix, Inning am Ammersee, Germany), the particle size/concentration and particle size distribution of the DPSC-derived apoVs were determined. Protein markers were identified using western blotting with antibodies against CD63, CD81, TSG101 (tumor susceptibility 101), and glyceraldehyde-3-phosphate dehydrogenase (GAPDH).

### 2.6. Cell Proliferation Assay

On 96-well plates, 2 × 10^3^ BMMSCs were planted in each well. The cells were subsequently grown in PM or PM containing 0.2, 0.6, 1.0, or 1.4 μg/mL apoVs, respectively. From day 0 to day 7, three wells of cells in the same treatment group were examined each day. Using a Cell Counting Kit-8 (CCK-8; Dojindo, Kumamoto, Japan), the cells were counted in accordance with the manufacturer’s instructions, and growth curves for the cells were obtained based on the cell count.

### 2.7. Alkaline Phosphatase (ALP) Staining and Quantification 

MSCs were sown in 12-well cell culture plates and cultivated with PM or OM for 4 or 7 days. MSCs were fixed for 30 min in 95% cold ethanol, and then rinsed three times with PBS. Following that, the manufacturer’s recommended procedures for ALP staining and quantification were followed (Nanjing Jiancheng Bioengineering Institute, Nanjing, China). Images with ALP staining were scanned. ALP was quantified by determining the absorbance at 520 nm and was normalized to the concentration of total protein.

### 2.8. Alizarin Red S (ARS) Staining and Quantification

In 12-well cell culture plates, the same starting density of MSCs as in Section 2.7 was seeded. Cells were gently rinsed with PBS after 7 or 14 days of growth in PM, OM, or OM with various doses of apoVs, fixed with 95% cold ethanol, and then washed with Milli Q water. Following that, ARS solution (2%, pH 4.2) was incubated with the cells. A 100 mM cetylpyridine solution was applied to the wells of a multiwell plate to measure mineral buildup. When the cells had completely dissolved, the absorbance at 562 nm was measured to determine the amount of mineral buildup.

### 2.9. RNA Extraction and Quantitative Real-Time Reverse Transcription PCR (qRT-PCR) 

Using the Trizol Reagent (Invitrogen, Waltham, MA, USA), total RNA was extracted from the cells; only RNA with an optical density ratio of 1.8 to 2.0 (260/280 nm) was suitable for reverse transcription into complementary DNA (cDNA), which was accomplished using a PrimeScript™ RT Reagent Kit (Takara, Dalian, China). Thereafter, using an SYBR Premix Ex Taq II Kit (Roche, Basel, Switzerland) and a Real-Time System, the quantitative real-time PCR (qPCR) step of the qRT-PCR protocol was carried out. The following thermal settings were employed: 40 cycles of 95 °C for 15 s and 60 °C for 1 min; 95 °C for 10 min. Table 1 contains a list of the primers used to amplify encoding osteocalcin (*OCN*), encoding alkaline phosphatase (*ALP*), encoding collagen type I alpha 1 (*COL1A1*), encoding runt-related transcription factor 2 (*RUNX2*), and internal standard (*GAPDH*).

### 2.10. Western Blotting

Western blotting was carried out in accordance with a previous investigation [12]. Using the RIPA Lysis Buffer System (Santa Cruz Biotechnology, Santa Cruz, CA, USA) combined with protease and phosphatase inhibitors, whole lysates of cells or apoVs were created. The concentrations of the extracted proteins were measured using a BCA Protein Assay Kit. Protein samples were loaded in equal amounts onto SDS-PAGE gels (LabLead, Beijing, China) and then transferred onto polyvinylidene fluoride (PVDF; Millipore, Billerica, MA, USA) membranes. Following that, the membranes were blocked using 5% non-fat milk in Tris-buffered saline 0.1% Tween 20 (TBST) for 3 h at room temperature. The primary antibodies were then applied to the membranes, followed by incubation at 4 °C overnight. The membranes were then treated with peroxidase-conjugated secondary antibodies for 1 h at room temperature after being rinsed with TBST. An ECL kit (CoWin Biotech, Taizhou, China) was utilized to detect the immunoreactive protein bands.

### 2.11. Heterotypic Bone Formation Assay In Vivo

Before in vivo implantation, MSCs cocultured with apoVs in PM were created. The cells were collected and combined with β-tricalcium phosphate (β-TCP; Bangalore, India) scaffolds for 1 h at 37 °C. Next, the mixtures were centrifuged at 300× *g* for 5 min. Six-week-old BALB/c homozygous nude (nu/nu) mice (n = 18 per group) were subcutaneously implanted with the scaffolds beneath the dorsal space. Each mouse had correspondent transplantation sites that were prepared at random for the transplantation of three groups of cells: β-TCP, β-TCP + PM, and β-TCP + PM/apoVs (n = 18 per group). Specimens were taken out and fixed in 4% paraformaldehyde for additional assays at 8 weeks after surgery. After decalcification for 14 days 10% EDTA (pH 7.2), the materials were dehydrated, embedded in paraffin, and sectioned at 4–5 μm thick. Hematoxylin and eosin (H&E) and Masson staining were carried out on the paraffin sections. All in vivo experiments were carried out in compliance with institutional animal guidelines and were approved by the Peking University Health Science Center’s Institutional Animal Care and Use Committee (approval number: LA 2023017).

### 2.12. In Vivo Experiment with apoV Injection

Ovariectomy (OVX) or SHAM procedures were performed on female ICR mice (7 weeks old; n = 36). Three groups of 18 mice were created at random: SHAM (n =12), OVX (n = 24). The OVX group mice underwent bilateral oophorectomy as per conventional procedures to create a mouse model simulating estrogen-deficiency-induced bone loss. The OVX mice were then divided into two groups at random: ovariectomy (OVX, PBS treatment) and ovariectomy, apoVs therapy (OVX + apoVs) (n = 12 per group).

Female mice aged 18 months were divided into two groups at random: (1) the aged + PBS group (n = 12) and (2) the aged + apoVs group (n = 12). A tail intravenous injection of apoVs dissolved in PBS was used to induce both the aged + apoVs group and the OVX + apoVs group (3 months after bilateral oophorectomy). Mice were anesthetized and then sacrificed after apoV injection once a week for 2 months. To get rid of the soft tissue, the femurs were dissected. The specimens were fixed in 4% paraformaldehyde for 24 h.

Ten days and three days prior to their demise, the mice received intraperitoneal injections of calcein and alizarin-3-methyliminodiacetic acid. The euthanized mice’s femur was taken out and repaired. Bioquant 2014 software (BIOQUANT Image Analysis Corporation, Nashville, TN, USA) was used to calculate the MAR.

### 2.13. Microcomputed Tomography and Bone Morphometric Analysis

Microcomputed tomography (micro-CT) was used to assess the variations in bone mass and microarchitecture between the groups. The images were analyzed quantitatively using software from Siemens’ Inveon Research Workspace (Siemens, Germany), which included measures of bone volume/total volume (BV/TV), trabecular number (Tb. N), trabecular separation (Tb. Sp), trabecular thickness (Tb. Th), bone surface area/bone volume (BS/BV), and bone mineral density (BMD).

The samples underwent 14 days of decalcification in 10% EDTA (pH 7.2), followed by dehydration and paraffin infiltration. Sections of 4–5 μm thickness were cut and then subjected to H&E and Masson staining. 

### 2.14. Osteogenic Efficiency in Bone Defects

According to our previous study, poly (lactic-co-glycolic acid) (PLGA) scaffolds modified with polydopamine (pDA) were constructed as the carriers of DPSC-derived apoVs [16]. In brief, PLGA (lactide/glycolide: 50/50) scaffolds purchased from the Shandong Academy of Pharmaceutical Sciences (Shandong, China) were prepared as cylinders of 5 mm in diameter and 2 mm in height. We soaked the PLGA scaffolds in dopamine (DA) solution (2 mg/mL in 10 mM Tris–HCl, pH 8.5) to form the pDA film. Next, the scaffolds were washed in PBS, subjected to sterilization with 75% ethanol, and rewashed. For the immobilization of the DPSC-derived apoVs, the PLGA scaffolds and PLGA/pDA scaffolds were incubated with DPSC-derived apoVs at 4 °C for 12 h. To observe the surface morphology of the scaffolds, PLGA, PLGA/pDA, and PLAG/pDA with apoVs were fixed in 4% glutaraldehyde for 12 h at 4 °C. All scaffolds were then dried in a critical point dryer. Images were then captured using an SU-8010 scanning electron microscope (SEM; Hitachi).

According to the guidelines established by the Experimental Animal Ethics Committee, the Peking University Health Science Center approved the animal assay (approval number: LA 2023017). The following three groups of 30 male Sprague-Dawley rats (n = 10 per group), aged 8 weeks, were randomly assigned: blank control (bone defects without any material), PLGA/pDA (PLGA framework overlaid with pDA) and PLGA/pDA + apoVs [16,28,29]. General anesthesia was administered via intraperitoneal injection using sodium pentobarbital (50 mg/kg). Low-speed drilling was used to create a 5 mm diameter critical-sized flaw at the calvarium. To prevent harm to the dura and brain, a significant saline flush, which lowers the temperature, was required. Thereafter, the scaffolds were inserted into the defects. After the wound was finally closed, all the rats were cultured in an environment-controlled animal care facility.

Eight weeks after surgery, all rats were euthanized under excessive anesthesia and sacrificed. The entire calvaria, including the implants, was carefully removed and placed in 4% paraformaldehyde. The growth of bone within the bone defect was imaged using a high-resolution Inveon Micro-CT. The specimens were subsequently decalcified in 10% EDTA for 21 days at pH 7.2. The decalcified specimens were embedded in paraffin and subjected to H&E and Masson staining.

### 2.15. Statistical Analysis

Utilizing GraphPad Prism 9.0 (GraphPad Inc., La Jolla, CA, USA), statistical and graph analysis were carried out. Unpaired Student’s t tests were used to determine the significance of two-group comparisons. By using one-way ANOVA and Tukey’s post hoc test, differences between several groups were examined. Using the Gehan–Breslow–Wilcoxon test, the survival rate was examined. Data are displayed as the mean ± standard deviation (SD). Values of *p* < 0.05 were considered statistically significant.

## 3. Results

### 3.1. Characterization of DPSC-Derived apoVs and the Uptake by MSCs

**Figure 1:**
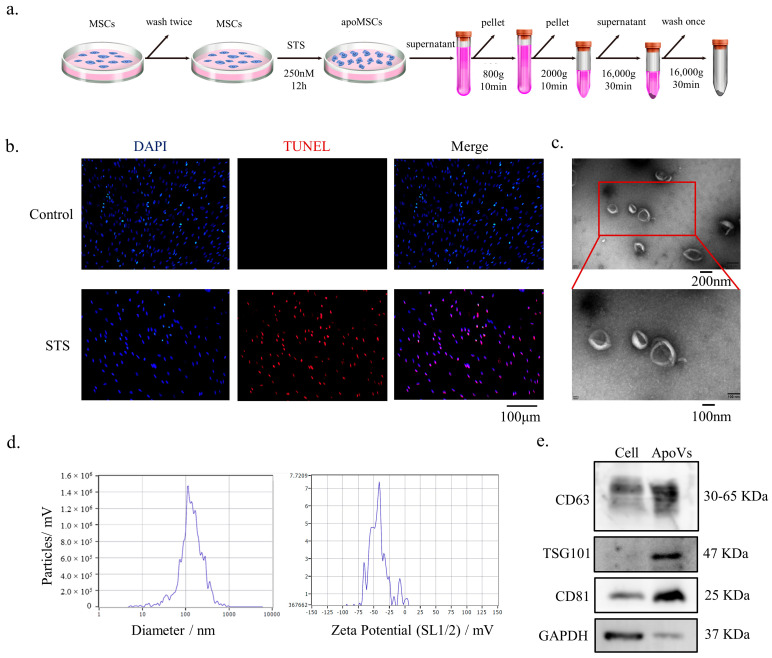
Characterization of DPSC-derived apoVs. (**a**) Schematic diagram indicating the procedures to isolate apoVs. (**b**) TUNEL staining results in the different groups. TUNEL positive stained cells are red. (**c**) Morphology of apoVs according to TEM analysis. (**d**) The size distribution and potential of the apoVs measured using nanoparticle tracking analysis. (**e**) Western blotting analysis of the DPSCs and DPSC-derived apoVs. STS, staurosporine; TEM, transmission electron microscopy; DPSC, dental pulp stem cell; apoVs, apoptotic vesicles; TUNEL, TdT-mediated dUTP nick end labeling; MSC, mesenchymal stem cell; DAPI, 4′,6-diamidino-2-phenylindole; CD36, CD36 molecule; TSG101, tumor susceptibility 101; CD81, CD81 molecule; GAPDH, glyceraldehyde-3-phosphate dehydrogenase.

MSC apoptosis was carried out as previously reported [26,30]. After DPSCs were washed twice with 0.22 μm filtered PBS, the medium was changed to 250 nM STS and α-MEM. ApoVs were extracted utilizing an improved gradient centrifugation procedure from apoptotic DPSCs (Figure 1a). Under the fluorescent microscope, both normal DPSCs and apoptotic DPSCs were observed. The STS group showed more positively stained cells (red) than the control group (Figure 1b). TEM analysis revealed that apoVs had a diameter of around 200 nm and a cup-shaped morphology (Figure 1c). ZetaView was used to determine the membrane potential and diameter distribution of the apoVs (Figure 1d). According to western blotting analysis, apoVs had high levels of CD63, CD81, and TSG 101 (Figure 1e).

### 3.2. ApoVs’ Internalization by MSCs

**Figure 2:**
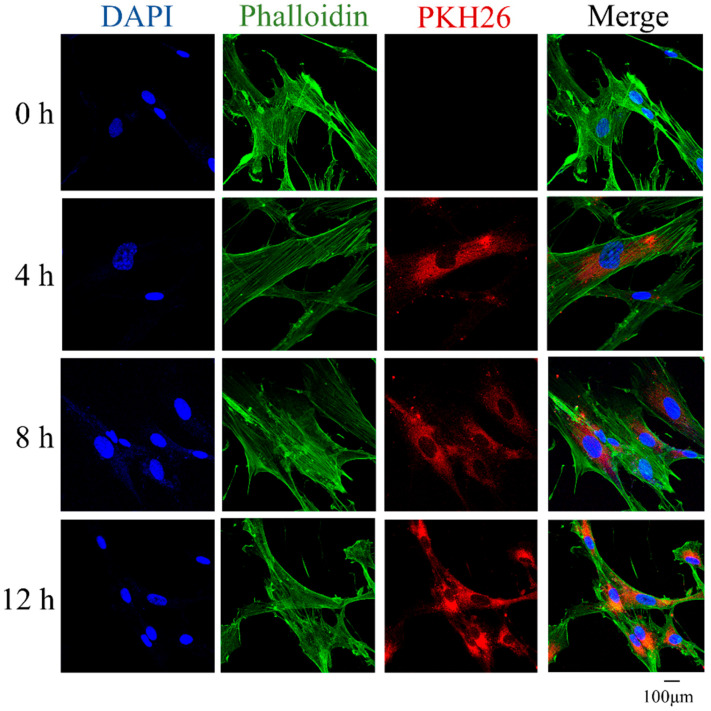
MSCs were incubated with PKH-26-labeled apoVs (red) for 4 h, 8 h, and 12 h, respectively. The nuclei of MSCs were stained with DAPI (blue). The F-actin of MSCs was stained with phalloidin (green). DAPI, 6-diamidine-2-phenylindole; MSCs, mesenchymal stem cells.

MSCs were treated with apoVs stained by PKH-26 (red) for 4, 8, and 12 h to determine if they could internalize them. Phalloidin was used to identify the F-actin in MSCs (green); DAPI (blue) was used to mark the nuclei. Images acquired under the confocal laser microscope showed that apoVs (red fluorescence) began to build up inside the MSCs and their number increased over the course of 4, 8, and 12 h. After incubation, a sizable number of apoVs were found in the intracellular zone (Figure 2).

### 3.3. ApoVs Promote the Osteogenic Differentiation of MSCs In Vitro and In Vivo

**Figure A1:**
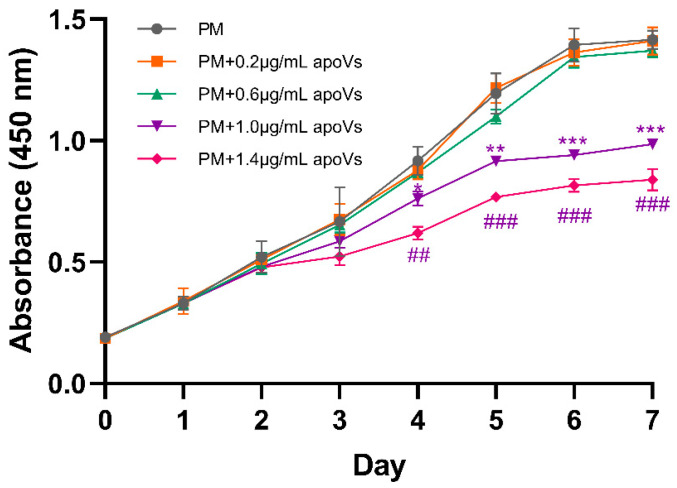
Growth curve measured using the Cell Counting Kit-8 (CCK-8) assay; apoVs at 1.0 μg/mL and 1.4 μg/mL inhibited the cell proliferation of MSCs from day 4 to day 7. * *p* < 0.05; ** *p* < 0.01; *** *p* < 0.001. ^##^ *p* < 0.01; ^###^ *p* < 0.001.

**Figure A2:**
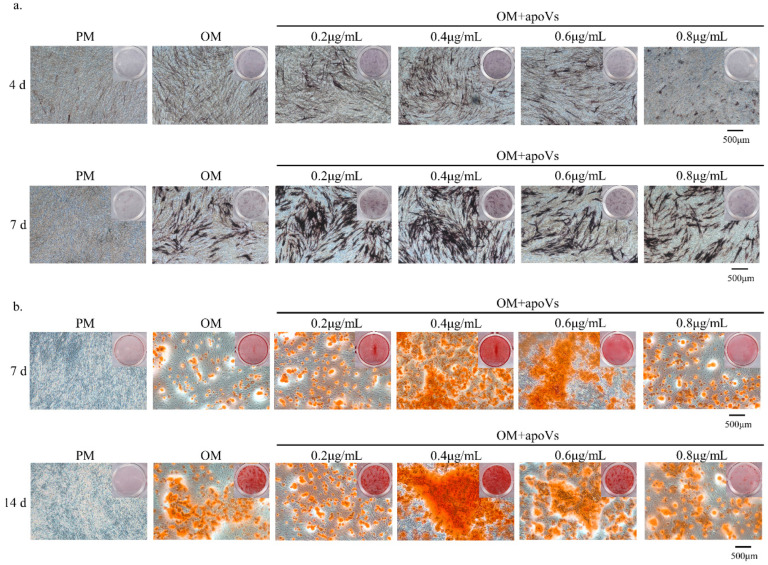
(**a**) ApoVs at 0.2, 0.4, and 0.6 μg/mL accelerated mineralization in MSCs, as indicated by ALP staining. ApoVs at 0.4 μg/mL showed the most gray-black massive and strip-shaped precipitation. (**b**) ApoVs at 0.2, 0.4, and 0.6 μg/mL accelerated mineralization in MSCs, as indicated by ARS staining. ApoVs at 0.4 μg/mL had the most mineralized nodule formation.

To determine a suitable or effective concentration range of apoVs, the CCK-8 assay was used to calculate the concentration range for the addition of apoVs. Several concentrations of apoVs were added to the medium (Figure A1, Appendix A). Accordingly, the optimal dose of apoVs to promote MSC osteogenesis was 0.4 μg/mL (Figure A2, Appendix A).

**Figure 3:**
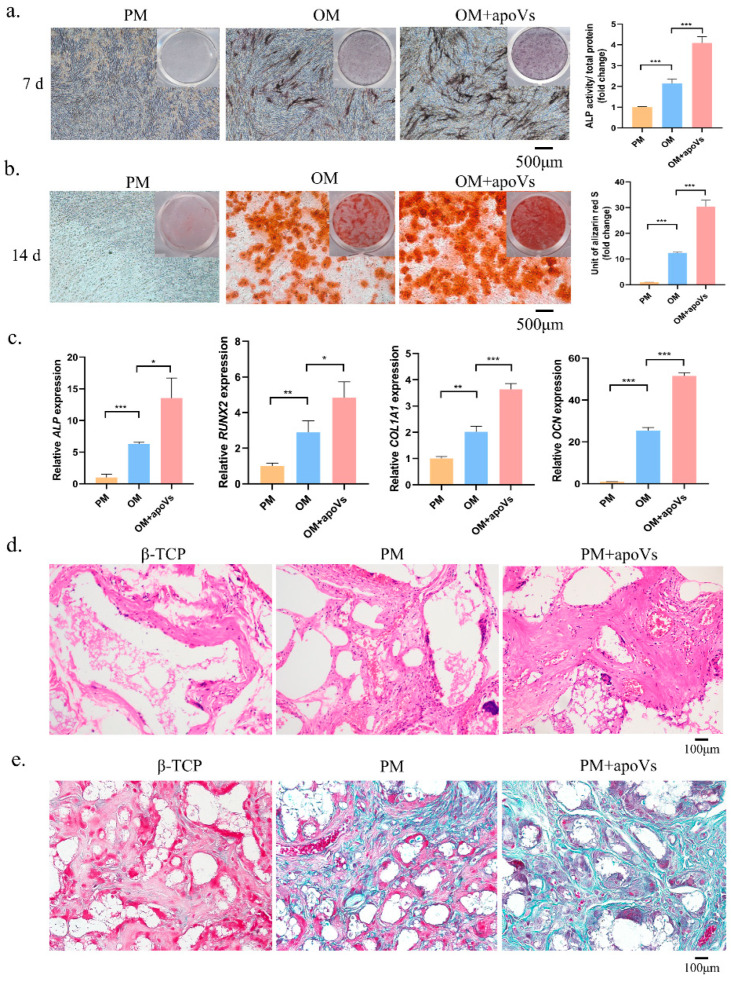
DPSC-derived apoVs enhanced the osteogenic differentiation of MSCs in vitro. (**a**) ApoVs increased ALP staining (left) and activity (right) on the seventh day after the osteogenic induction of MSCs. (**b**) ApoVs promoted ARS staining (left) and quantification (right) on the 14th day after the osteogenic induction of MSCs. (**c**) ApoVs promoted the mRNA expression of *ALP*, *RUNX2*, *OCN,* and *COL 1A1* detected by RT-qPCR. (**d**) The neo-generated tissues were characterized by H&E staining. (**e**) Masson staining of histological sections. All data are shown as the mean ± SD. * *p* < 0.05, ** *p* < 0.01 and *** *p* < 0.001. ALP, alkaline phosphatase; ARS, alizarin red S; OM, osteogenic media; PM, proliferation media; H&E, haematoxylin-eosin; β-TCP, β-tricalcium phosphate; *RUNX2*, runt-related transcription factor 2; *OCN*, osteocalcin; *COL1A1*, collagen type I alpha 1 chain; qRT-PCR, quantitative real-time reverse transcription PCR.

ApoVs had a considerable impact on the promotion of osteogenic differentiation, as demonstrated by ALP and ARS staining (Figure 3a,b). Additionally, compared with that in the OM group, MSCs treated with apoVs had significantly higher *OCN*, *RUNX2*, *ALP*, and *COL1A1* mRNA expression levels (Figure 3c). Next, we combined MSCs cultivated in PM and PM + apoVs with β-TCP and implanted the mixture into nude mice to ascertain the function of apoVs in MSC osteogenesis in vivo. H&E staining revealed that the PM + apoVs group showed highly eosinophilic tissue and more characteristics that were similar to bone tissue than the β-TCP and PM groups (Figure 3d). There were fewer blue-green collagen fibers in the β-TCP group than in the PM group, whereas the apoVs + PM group had the highest density, as demonstrated by Masson staining. These findings demonstrated that apoVs promoted MSC osteogenesis in vivo (Figure 3e).

### 3.4. ApoVs Reduced the Loss of Microarchitecture and Bone Mass in OVX Mice

Osteoporosis is a systemic bone disease marked by a low bone mass and degeneration of the bone microstructure, increasing the susceptibility of the bone to fractures [31,32]. The most common osteoporosis is premenopausal osteoporosis and senile osteoporosis. Therefore, we investigated how DPSC-derived apoVs affected bone loss in ovariectomized (OVX) mice and aged mice.

**Figure A3:**
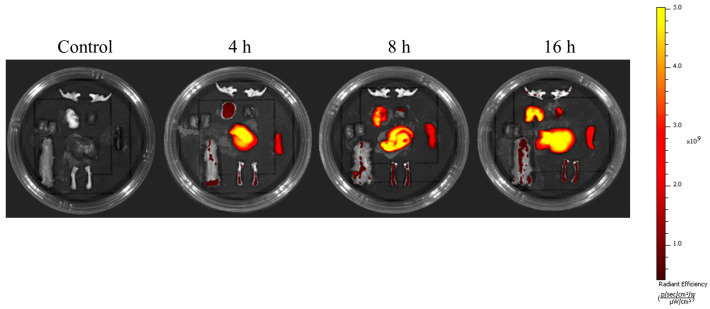
Reprehensive time-dependent in vivo fluorescence images of apoVs with DiIC18(7) (1,1′-Dioctadecyl-3,3,3′,3′-Tetramethylindotricarbocyanine Iodide (DiR) dyes in mice. The signals were collected in the 780 nm channel with laser excitation at 750 nm.

Understanding the biodistribution of apoVs is especially crucial when designing apoVs for therapeutic applications. We first examined the distribution of DiIC18(7) (1,1′-Dioctadecyl-3,3,3′,3′-Tetramethylindotricarbocyanine Iodide (DiR)-apoVs after injecting them into the tail veins of mice. ApoVs were detected in the lungs, liver, spleen, and bone, suggesting that apoVs could reach the bones (Figure A3, Appendix A).

**Figure 4:**
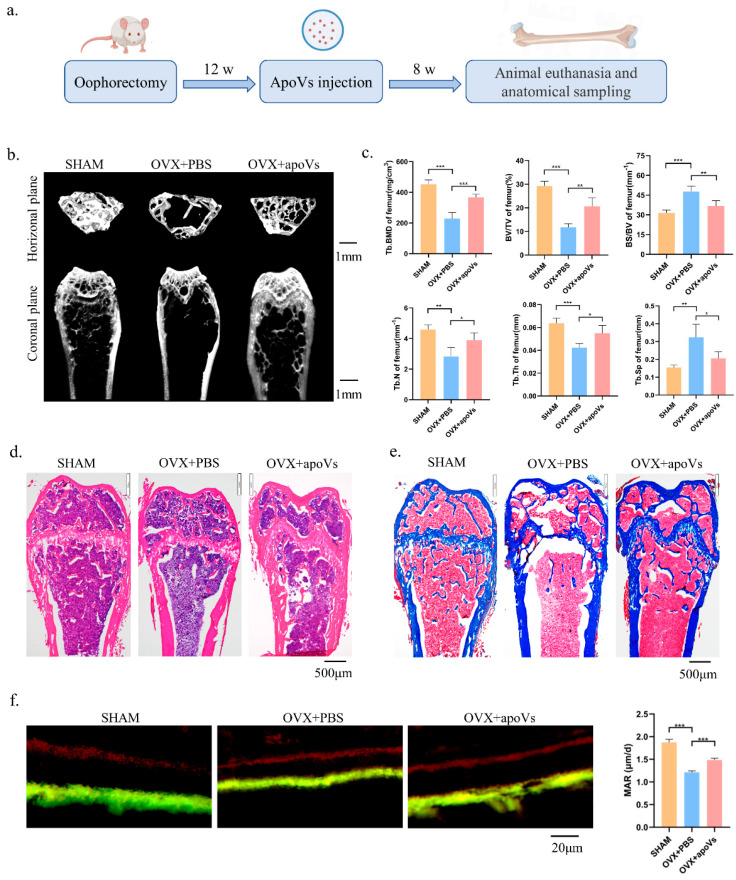
ApoVs attenuated the bone loss induced by estrogen deficiency in OVX mice. (**a**) The osteoporosis mouse models were established by removing both ovaries, followed by tail vein injection of apoVs once a week for 8 weeks. (**b**) Micro-CT images of femurs in the SHAM mice with PBS treatment, OVX mice with PBS treatment, and OVX mice with apoVs treatment. (**c**) Quantitative measurements of BV/TV, Tb. N, Tb. Sp, Tb. Th, BS/BV, and BMD of the SHAM and OVX groups. (**d**) H&E staining. (**e**) Masson staining. (**f**) Representative images of new bone formation in the distal femoral epiphysis, assessed using double-labeling with calcein and alizarin-3-methyliminodiacetic acid. Dynamic MAR measured from the femur. All data shown are the mean ± SD. * *p* < 0.05, ** *p* < 0.01, and *** *p* < 0.001. MAR, mineral apposition rate; OVX, ovariectomized; micro-CT, Inveon micro-computed tomography; the Inveon Research Workplace 4.2 software, a three-dimensional reconstruction and parametric analysis; PBS, phosphate-buffered saline; BV/TV, bone volume/total volume; Tb. N, trabecular number; Tb. Sp, trabecular separation; Tb. Th, trabecular thickness; BS/BV, bone surface area/bone volume; BMD, bone mineral density.

We then provided once-weekly injections of apoVs into the experimental group’s tail veins, whereas the control group received injections of PBS based on the OVX mouse model, as illustrated in the schematic diagram. After eight tail vein injections, the experimental group was euthanized, and samples were obtained and various regions were examined (Figure 4a). The bone histomorphology of the femur was assessed using micro-CT to investigate the impact of apoVs on OVX-induced bone loss in mice. Figure 4b displays representative pictures of the femur. The BMD, BV/TV, Tb. Th, and Tb. N values of the OVX + apoVs group were considerably higher than those of the OVX + PBS group (Figure 4c). Using H&E and Masson staining, it was discovered that, in the OVX mice, apoVs prevented the bone loss induced by estrogen deprivation (Figure 4d,e). When double fluorescent labeling was utilized to assess the dynamic histomorphometry, the mineral apposition rate (MAR) was higher in the OVX + apoVs group compared to the OVX + PBS group (Figure 4f).

### 3.5. ApoVs Reduced the Loss of Microarchitecture and Bone Mass in Aged Mice

**Figure 5:**
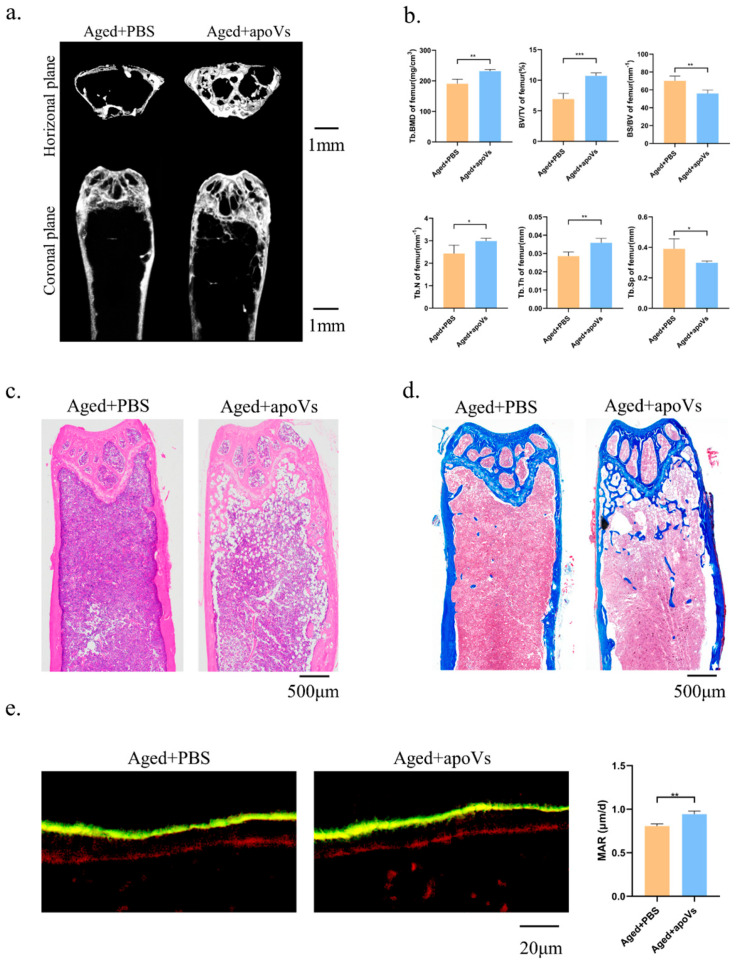
ApoVs partially reversed bone loss in aged mice. (**a**) Micro-CT images of femurs in aged mice treated with PBS and treated with apoVs for 8 weeks. (**b**) Quantitative measurements of BV/TV, Tb. N, Tb. Sp, Tb. Th, BS/BV, and BMD of aged groups. (**c**) H&E staining. (**d**) Masson staining. (**e**) Representative images of new bone formation in the distal femoral epiphysis assessed by double-labeling with calcein and alizarin-3-methyliminodiacetic acid. Dynamic MAR measured from the femur. All data shown are the mean ± SD. * *p* < 0.05, ** *p* < 0.01 and *** *p* < 0.001.

**Figure A4:**
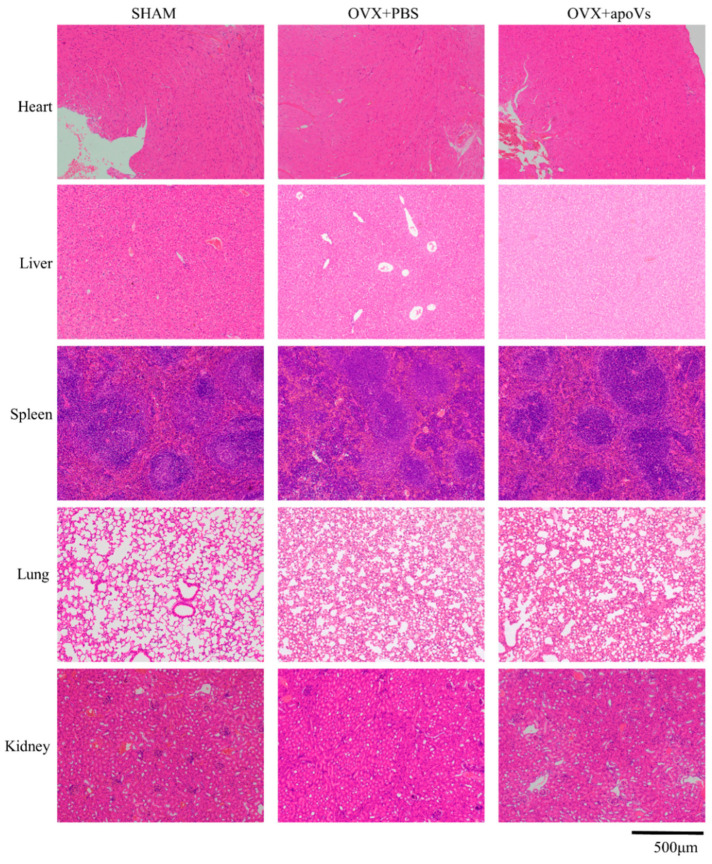
ApoVs caused no toxicity or inflammation in the viscera of the OVX mice. Tail vein injection of apoVs for 8 weeks did not cause toxicity or inflammation changes in the kidney, liver, spleen, lung, or heart of mice. Scale bar = 500 μm.

**Figure A5:**
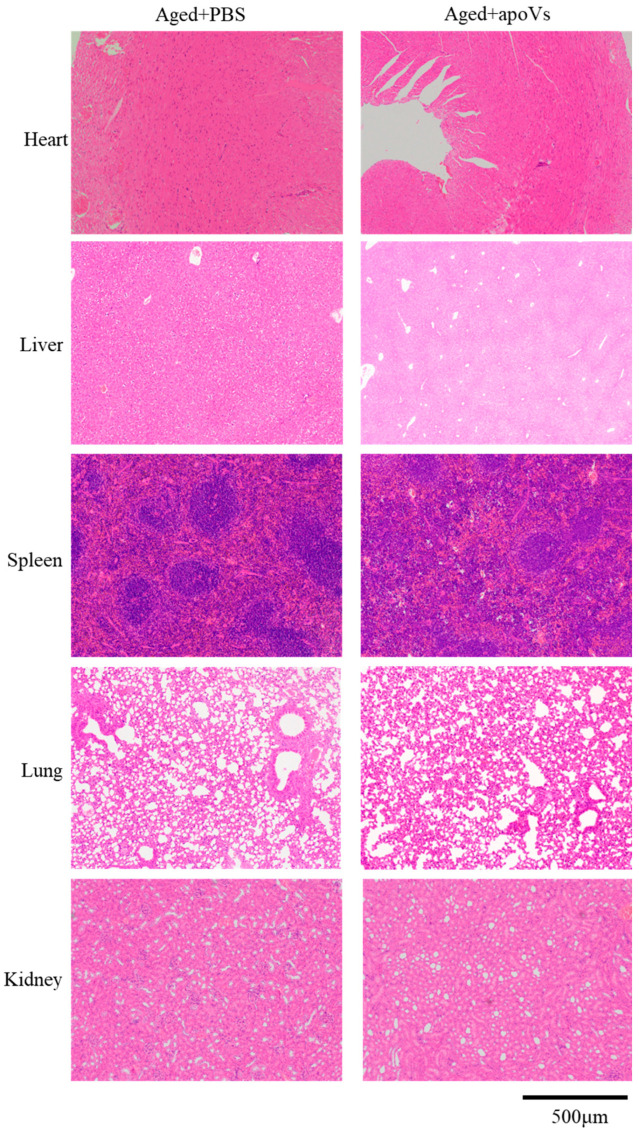
ApoVs caused no toxicity or inflammation in the viscera of the aged mice. Tail vein injection of apoVs for 8 weeks did not cause toxicity or inflammation changes in the kidney, liver, spleen, lung, or heart of mice. Scale bar = 500 μm.

Mice aged 18–30 months are suitable for the construction of the natural aging osteoporosis model, showing a decrease in bone density and bone mass [33,34]. The bone histomorphology of the femur of the aged mice was assessed using micro-CT to investigate the impact of apoVs on aging-induced bone loss (Figure 5a). Comparing the aged + apoVs group to the aged + PBS group, there were significant increases in femoral BV/TV, BMD, Tb. N, and Tb. Th, as well as significant decreases in BS/BV and Tb. Sp. (Figure 5b). The H&E and Masson staining of the aged mice revealed that the apoVs could prevent bone loss (Figure 5c, d). Double fluorescent labeling demonstrated that the aged + apoVs group exhibited a higher MAR in comparison to the aged + PBS group (Figure 5e). Additionally, liver, spleen, kidney, lung, and heart slices from the mice did not exhibit any indications of inflammatory infiltration (Figure A4 and Figure A5, Appendix A).

### 3.6. ApoVs Promoted Critical-Sized Rat Calvarial Defects In Vivo

To gain further insight into the potential therapeutic benefit of DPSC-derived apoVs, their role in bone defects in situ was evaluated. In our prior studies, we effectively combined and released extracellular vesicles (EVs) in mouse calvarial defects using polydopamine-coated PLGA scaffolds (PLGA/pDA) [26,35].

**Figure 6:**
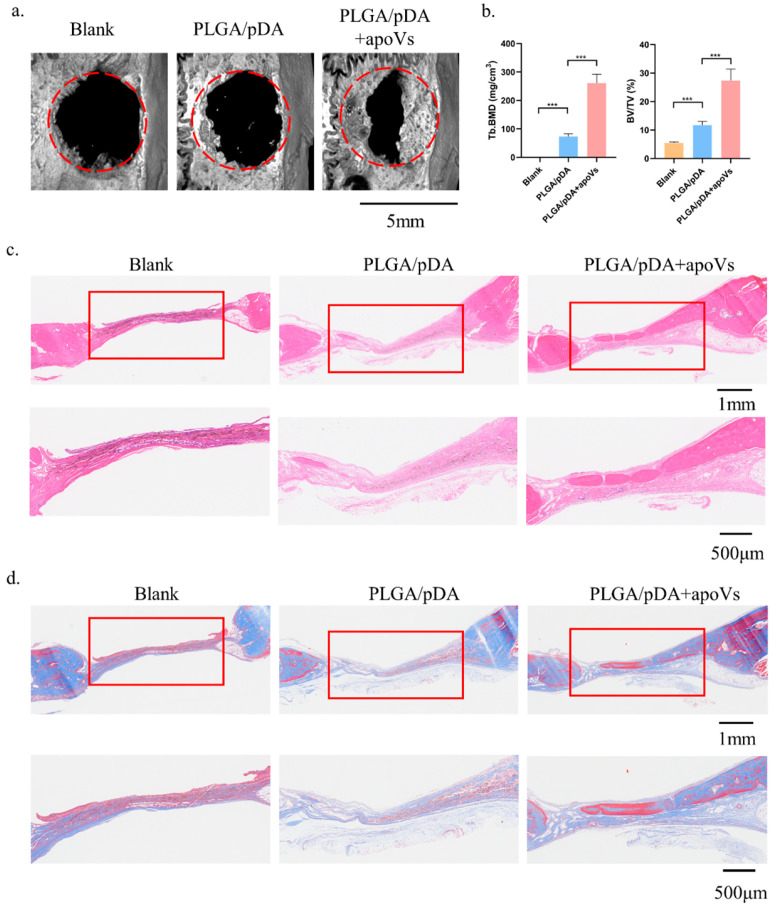
ApoVs increased bone formation in critical-sized rat calvarial defects. Rats were treated with PLGA scaffolds (PLGA), PLGA scaffolds with poly-dopamine coating (PLGA/pDA) or PLGA scaffolds coated with poly-dopamine and apoVs (PLGA/pDA + apoVs). (**a**) Micro-CT images of bone formation in each group after 6 weeks. (**b**) Quantitative comparison of new bone volume among the different groups. *** *p* < 0.001 compared with groups without apoVs. Histological assessment of bone formation in each group: (**c**) H&E staining. (**d**) Masson staining. PLGA, poly (lactic-co-glycolic acid); PLGA/pDA, PLGA scaffolds coated with polydopamine; micro-CT, Inveon micro-computed tomography; the Inveon Research Workplace 4.2 software, a three-dimensional reconstruction and parametric analysis.

**Figure A6:**
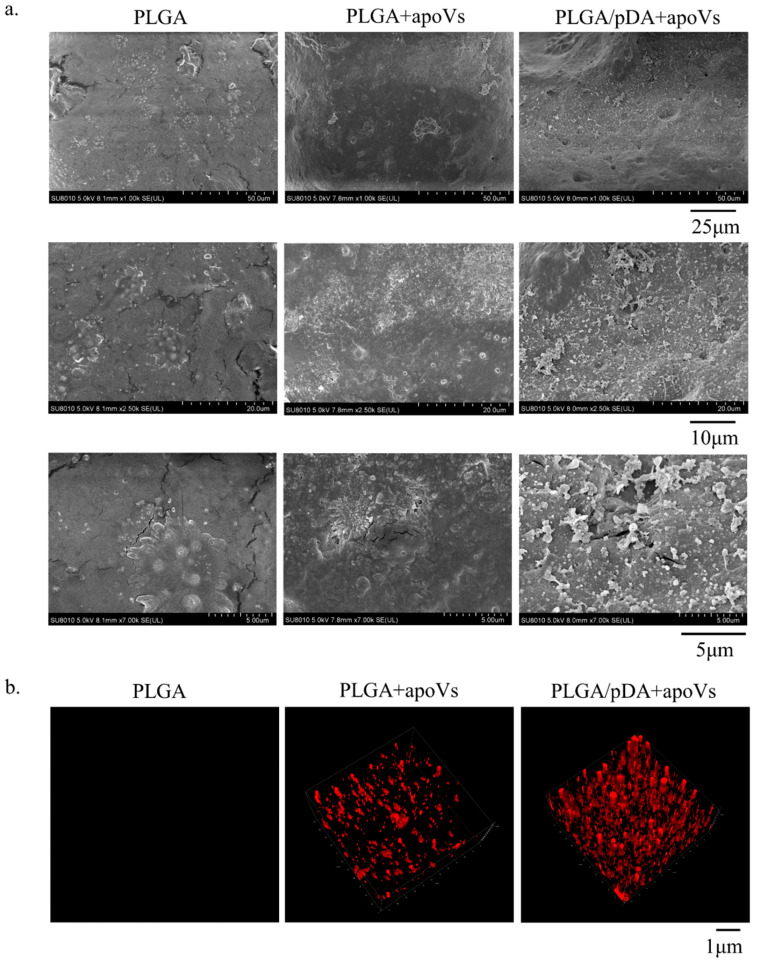
(**a**) Scanning electron microscopy of PLGA scaffolds (PLGA), PLGA scaffolds coated with poly-dopamine coating (PLGA/pDA) and PLGA scaffolds coated with poly-dopamine and apoVs (PLGA/pDA + apoVs). (**b**) Distribution of PKH-26 labeled apoVs on the PLGA/pDA scaffold, with PKH-26-stained PLGA scaffold as control.

In this study, we built a cell-free tissue-engineered bone constructed system using PLGA/pDA scaffolds in conjunction with apoVs. The apoVs particles dispersed on the surface of the PLGA/pDA + apoVs scaffold and had a ball-shaped and refractive morphology, in contrast to the polydopamine particles on the PLGA/pDA scaffold (Figure A6a, Appendix A). PKH-26-labeled apoVs (red dots) were uniformly distributed across the scaffold surface (Figure A6b, Appendix A). Then, rat models with critical-sized calvarial defects were created. A blank group was established, and the remaining two groups were implanted with either PLGA/pDA or PLGA/pDA with apoVs (PLGA/pDA + apoVs). The PLGA/pDA + apoVs group displayed high-density new bone at the defect boundaries, according to micro-CT images, while the PLGA/pDA group and the blank group showed almost no healing (Figure 6a). According to bone morphometric measurement, the PLGA/pDA + apoVs group had higher BMD and BV/TV values, which demonstrated that the apoVs significantly promoted bone formation (Figure 6b). H&E staining revealed only fibrotic tissue in the defects in the PLGA/pDA and blank groups, whereas the PLGA/pDA + apoVs group had more new bone tissue than the other groups (Figure 6c). Masson staining revealed that the PLGA/pDA + apoVs group had more blue-stained collagen structures than the other two groups (Figure 6d).

### 3.7. ApoVs Promote the Osteogenic Development of MSCs via the ERK1/2 Signaling Pathway

**Figure 7:**
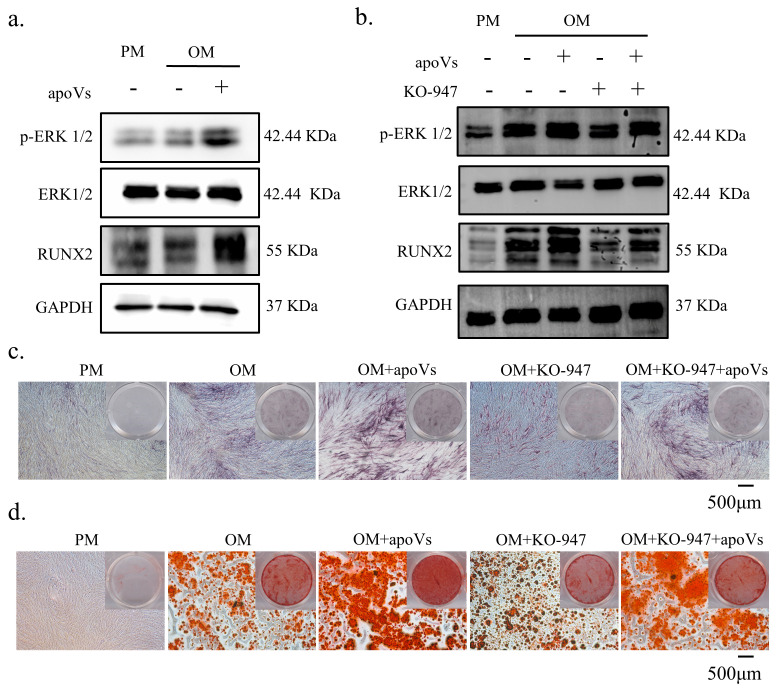
ApoVs regulated the osteogenic differentiation of MSCs via the ERK1/2 signaling pathway. (**a**) Western blotting of the protein expression of p-ERK, ERK, RUNX 2, and GAPDH. MSCs were treated with PM, OM, and OM + apoVs for 7 days. (**b**) Western blotting of the protein expression of p-ERK, ERK, RUNX 2, and GAPDH. MSCs were treated with PM, OM, OM + apoVs, OM + KO-947, and OM + KO-947 + apoVs for 7 days. ALP staining (**c**) and ARS staining (**d**) in the PM, OM, OM + apoVs, OM + KO-947, and OM + KO-947 + apoVs groups. KO-947, ERK1/2 signaling pathway inhibitor.

**Figure 8:**
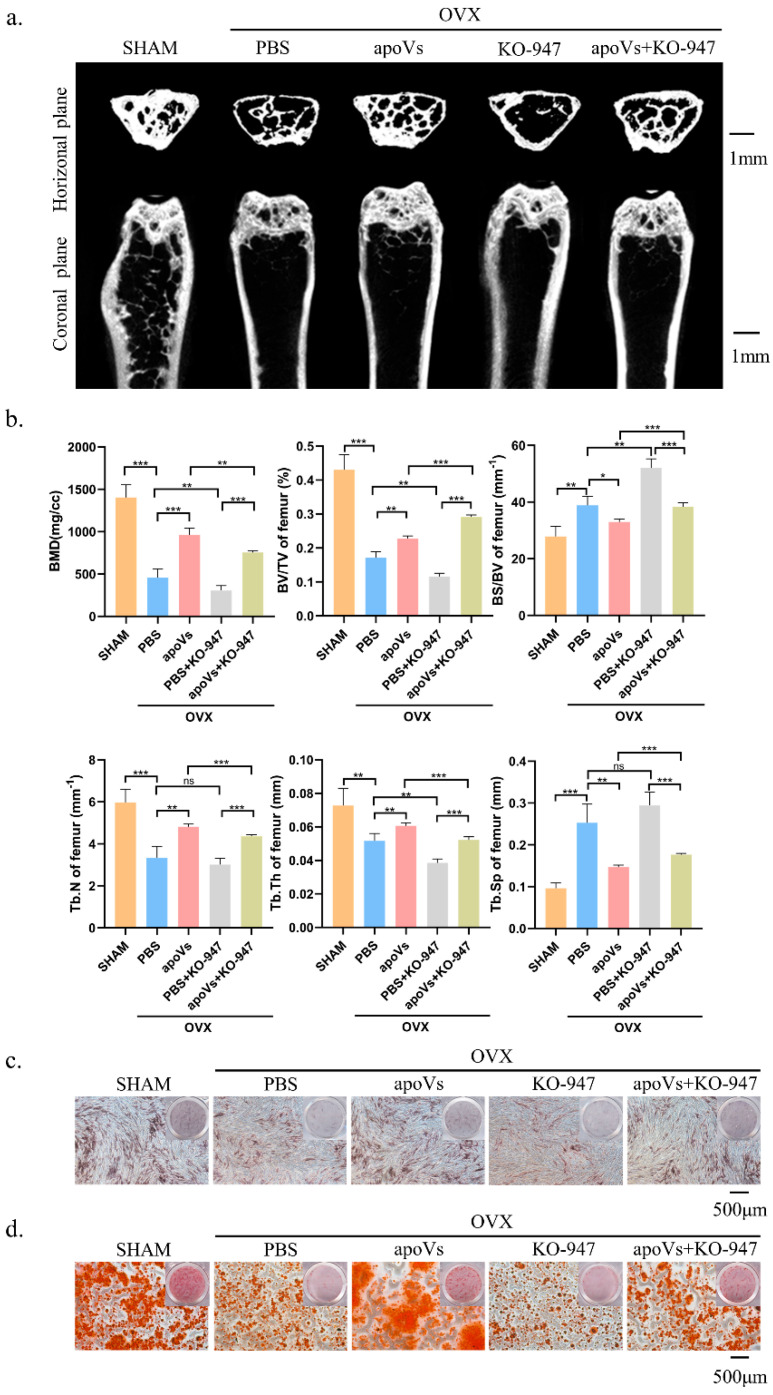
ApoVs can partially restore the effect of the ERK1/2 pathway inhibitor on bone loss in OVX mice. (**a**) Micro-CT images of femurs among the SHAM, OVX + PBS, OVX + apoVs, OVX + KO-947, and OVX + apoVs + KO-947 groups. (**b**) Bone morphometry analysis among these groups. Mouse bone marrow mesenchymal stem cells (mBMMSCs) extracted from mouse femurs from the different groups were stained (**c**) with ALP on day 7 and (**d**) with ARS on day 14. ns, no significance; * *p* < 0.05; ** *p* < 0.01; *** *p* < 0.001.

**Figure A7:**
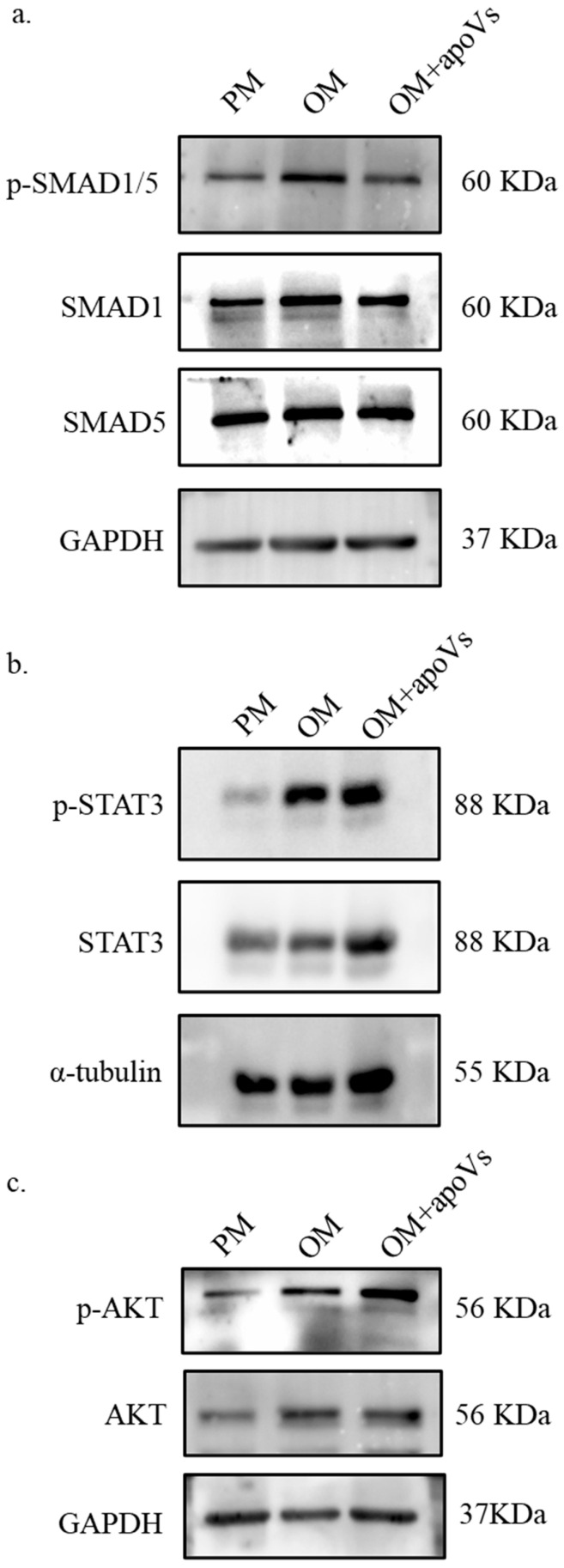
The alterations in MSC signaling pathways following the addition of apoVs. MSCs were treated with PM, OM, and OM + apoVs for 7 days. (**a**) Western blotting of the protein levels of phosphorylated (p)-SMAD1/5, SMAD1, SMAD5, and GAPDH. (**b**). Western blotting of protein expression of p-STAT3, STAT3, and α-tubulin. (**c**) Western blotting of protein expression of p-AKT, AKT and GAPDH. SMAD1, SMAD family member 1; SMAD5, SMAD family member 5; STAT3, signal transducer and activator of transcription 3; AKT, protein kinase B.

**Figure A8:**
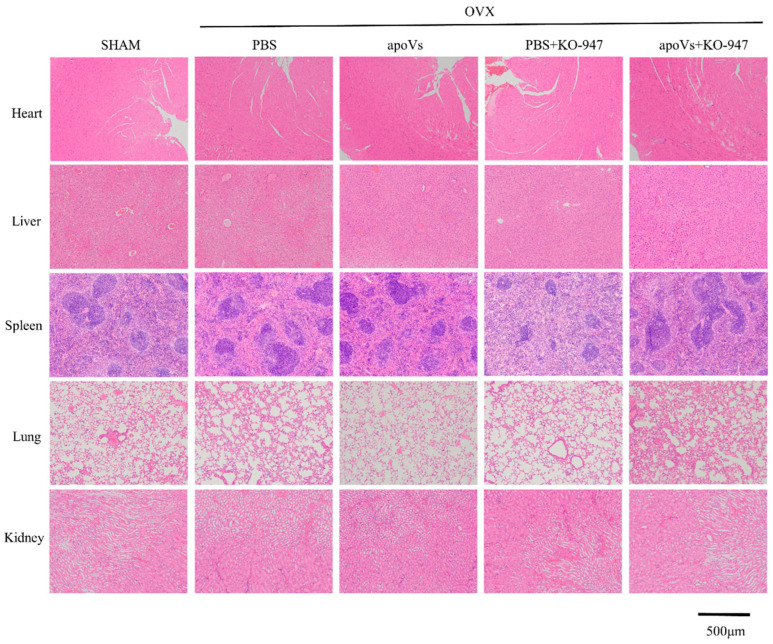
ApoVs and KO-947 caused no toxicity or inflammation in the viscera of the OVX mice. Tail vein injection of apoVs for 8 weeks did not cause toxicity or inflammation changes in the kidney, liver, spleen, lung, or heart of mice. Scale bar = 500 μm.

There are several signaling pathways that can affect osteogenic differentiation in MSCs, such as the JAK/STAT signaling pathway and the PI3K/AKT signaling pathway. Compared with other pathways, we found that DPSC-derived apoVs has the most obvious effect on promoting the osteogenic differentiation of MSCs through the ERK1/2 signaling pathway (Figure A7, Appendix A). The level of phosphorylated ERK1/2 was elevated following treatment with apoVs for 7 days, which prompted us to investigate the mechanism by which apoVs influence osteogenesis (Figure 7a). The results implied that apoVs could regulate the ERK1/2 pathway [35,36]. Hence, we treated MSCs with 10 nM KO-947, an inhibitor of the ERK1/2 signaling pathway [37]. Western blotting revealed that KO-947 decreased the levels of RUNX2 and phosphorylated ERK1/2, which was partially restored by apoVs therapy (Figure 7b). ALP staining and ARS staining showed that KO-947 inhibited the promotion of osteogenesis in MSCs via apoVs (Figure 7c,d). The OVX mice were given tail vein injections of PBS, apoVs, KO-947 (300 mg/kg), and KO-947 + apoVs, respectively. Using micro-CT, the histomorphology of the femur was assessed (Figure 8a). Compared with those in the KO-947 group, the KO-947 + apoVs group showed noticeably increased levels of BV/TV, BMD, Tb. N, and Tb. Th (Figure 8b). Furthermore, no signs of inflammatory infiltration were found in the mouse lung, heart, liver, spleen, or renal organ slices (Figure A8, Appendix A). Additionally, mouse bone marrow mesenchymal stem cells (mBMMSCs) isolated from mice femurs in the various groups were stained with ALP and ARS on day 7 and 14, respectively (Figure 8c, d). These findings imply that the therapeutic impact of apoVs on bone loss acts via the ERK1/2 signaling pathway.

## 4. Discussion

Adult stem cells, such as MSCs, exist in various tissues and have the potential for multidirectional differentiation. These types of stem cells are expected to be developed as useful cell carriers to achieve targeted drug delivery due to their natural inflammatory homing characteristics [38]. It is generally believed that the therapeutic effect of MSCs is closely related to their multi-directional differentiation potential and paracrine function. Therefore, maintaining the good biological activity of MSCs is very important for their therapeutic effects. However, some studies have shown that most exogenous MSCs, when injected into the body, will rapidly undergo apoptosis because of the poor microenvironment of the diseased tissues, making it impossible to further exert long-term therapeutic effects through cell differentiation and paracrine functions [15]. Therefore, researchers have speculated that there might be other potential mechanisms to achieve the therapeutic effect of MSCs. Recent studies have found that apoptotic MSCs also have therapeutic effects. During MSC apoptosis, apoVs are released, which have been proven to play an important role in the treatment of skin trauma, liver fibrosis, myocardial infarction, and other diseases [13,39,40]. Therefore, MSC-derived apoVs are expected to become a new therapeutic agent.

ApoVs are mostly composed of protein, nucleotides, lipids, and metabolites [41,42]. They are thought to be an evolutionarily conserved method of intercellular communication, sending signals from parent cells to the extracellular environment to control the activity of recipient cells [42]. According to recent studies, the processes of tissue regeneration and recovery from injury in the human body are directly linked to apoptosis. It was shown that EVs derived from BMMSCs can promote the migration, proliferation, and osteogenic differentiation of BMMSCs [43]. Moreover, research has demonstrated that mBMMSC-derived exosomes can promote the proliferation of osteoblasts through the MAPK signaling pathway, thus improving osteoporosis in mice [44]. Osteoclast-derived apoVs could act on osteoblasts and promote their osteogenic differentiation by activating the PI3K/AKT pathway [45]. ApoVs have also been proven to play a role in bone remodeling. For example, osteoblast-derived apoVs could recruit osteoclasts, leading to local bone resorption [46], while BMMSC-derived apoVs released miR1324 to activate the SMAD1/5 pathway to improve bone loss in osteoporotic mice [26].

DPSCs are a kind of ectodermal MSCs that show self-renewal, multi-directional differentiation, and high proliferation. These cells exist in the pulp of permanent teeth/deciduous teeth and are the most active mesenchymal stem cells, with three times the activity of BMMSCs [47]. A small amount of DPSCs can divide and proliferate into tens of millions of daughter cells, demonstrating a very strong differentiation ability. They can differentiate into osteoblasts, adipocytes, hepatocytes, and other human cells depending on whether there is a suitable in vivo or in vitro environment, thus providing a new cell source for tissue and organ repair and transplantation [18].

In this study, we uncovered the roles of DPSC-derived apoVs and their potential mechanisms in bone regeneration. We showed that apoptotic DPSCs secrete apoVs, which are cup-shaped and less than 1 μm in diameter. DPSC-derived apoVs could be taken up by MSCs. Both in vitro and in vivo, DPSC-derived apoVs could promote MSC osteogenesis. Furthermore, apoVs prevented bone loss in osteoporotic mice and promoted bone regeneration in rat cranial defects. Our findings thus demonstrated the importance of DPSC-derived apoVs in promoting MSC differentiation into osteoblasts and suggested that they could be a viable treatment for both localized and systemic bone loss.

Bone homeostasis depends on a bone remodeling process in which bone resorption by osteoclasts and bone formation by osteoblasts are tightly coupled and dynamically balanced [48,49]. Osteoblasts differentiated from MSCs are responsible for bone formation and osteoclasts differentiated from monocytes are responsible for bone resorption, and these two types of cells maintain the balance between bone formation and resorption in bone homeostasis [50,51]. When the dynamic balance between bone resorption and formation is disturbed, it will cause an imbalance in bone homeostasis, leading to a variety of diseases, such as osteosclerosis and osteoporosis. Osteoporosis, a degenerative disease, has emerged as a major public health concern in the aging population [52]. 

Current therapeutic strategies for osteoporosis focus on inhibiting osteoclast activity or inducing bone formation [53,54]. For many decades, selective estrogen receptor modulators (SERMs), bisphosphonates, denosumab, calcitonin, and estrogens have been used in clinical practice as inhibitors of osteoclast activation and differentiation [55,56]. However, these drugs are unable to restore osteoblast function and reconstruct the bone microarchitecture, and might also cause adverse effects [57]. Although autologous bone transplantation is the gold standard for the reconstruction of large bone defects, it also has some shortcomings, such as a lack of sufficient transplantable materials, donor site disease, bone resorption, and inflammation [58]. 

Previous studies have demonstrated that MSCs could increase bone formation, resulting in their wide use in osteoporosis treatments [59,60,61]. As a kind of acellular preparation, the transplantation of DPSC-derived apoVs has certain advantages, including low immunogenicity, easy storage, and a reduced risk of coagulation. Our study found that the systemic intravenous infusion of DPSC-derived apoVs could slow down osteoporosis, and the local implantation of DPSC-derived apoVs could promote the healing of bone defects. In addition, DPSC-derived apoVs can promote the osteogenic differentiation of MSCs, increase the number of osteoblasts, and significantly promote bone regeneration. 

Exploring the mechanism of the osteogenic differentiation of MSCs can provide new ideas for efficiently initiating their osteogenic differentiation, and will provide a new theoretical basis for the treatment of bone defects. The ERK1/2, PI3K/AKT, and SMAD pathways are common molecular pathways that regulate MSC osteogenic differentiation [62,63]. According to our findings, DPSC-derived apoVs can encourage MSCs to differentiate into osteoblasts, and p-ERK1/2 is a key player in this process. By augmenting ERK1/2 phosphorylation, apoVs stimulated the ERK1/2 signaling pathway and controlled bone homeostasis. The ERK1/2 pathway inhibitor KO-947 was added to cells and to the animal trials to prevent MSCs from differentiating into osteoblasts; however, apoVs could alleviate its inhibitory effect. ERK1/2 has a typical protein kinase structure and regulates cell activities by phosphorylating substrates [35,64]. Extracellular signals such as cytokines, hormones, and cell stress enter the cell through transmembrane receptors and are transmitted to the cell along the RAF-MEK-ERK signaling pathway. In this process, ERK1/2 is the only substrate of MEK; however, it can phosphorylate hundreds of downstream cytoplasmic and nuclear substrates, thus playing an important role in the transmission of extracellular signals to cells. Our findings demonstrated that DPSC-derived apoVs promote bone regeneration through the ERK1/2 signaling pathway; however, the underlying mechanism still needs further in-depth study. Other bioactive substances, such as microRNAs, might also contribute to the therapeutic effect of DPSC-derived apoVs.

Nonetheless, there are still some problems or challenges in the application of apoVs for disease treatment. Firstly, our experiment only studied small animals; thus, in vivo data of large animals, such as miniature pigs and dogs, are required in the future. Secondly, engineered apoVs could be further designed to show increased bone targeting, allowing for them to be applied to the whole body and guide bone regeneration. Thirdly, a better local application method is required. For example, we previously demonstrated that hydrogel can be combined with freeze-dried apoVs to further enhance their local bone induction efficiency and enhance their osteogenic effect [65]. Despite these challenges and uncertainties, we still believe in the potential and value of DPSC-derived apoVs in future clinical bone regeneration. In mass production, considering the requirement for establishing large-scale cell culture technology, DPSC-derived apoVs will become a competitive cell-free preparation with a high yield advantage as long as the developed induction method is applied. Furthermore, the separation and purification technology of apoVs is gradually being optimized. Currently, the exploration and application of DPSC-derived apoVs in bone regeneration is still at the early stage; therefore, more in-depth research is needed, such as modifying apoVs to overcome the problems of premature removal and complex content, thereby promoting the application of apoVs.

## 5. Conclusions

In this study, we found that DPSC-derived apoVs could significantly promote the osteogenic differentiation of MSCs in vivo and in vitro. In addition, DPSC-derived apoVs could slow down the bone loss in osteoporosis mice and promote the growth of local defect bone, which provides a new direction to further explore the therapeutic effect of DPSC-derived apoVs in promoting bone regeneration systemically and locally. Mechanically, DPSC-derived apoVs can regulate osteogenic differentiation and enhance ERK1/2 phosphorylation in MSCs via modulating the ERK1/2 signaling pathway. This finding lays the foundation for the potential clinical use of DPSC-derived apoVs. Information regarding the regulatory function and underlying mechanisms of apoVs will help us to better understand bone regeneration and repair, as well as creating novel treatment strategies to treat bone loss and defects. DPSC-derived apoVs offer a promising strategy to prevent bone loss and promote cell-free bone tissue engineering because of their ease of production and storage, low risk of immunological rejection, high stability, and their capacity to target specific cells and tissues.

## Figures and Tables

**Table 1:** List of primers used in this study.

Gene	Forward Primer (5′-3′)	Reverse Primer (5′-3′)
*OCN*	AGCCACCGAGACACCATGAGA	GGCTGCACCTTTGCTGGACT
*RUNX2*	ACTACCAGCCACCGAGACCA	ACTGCTTGCAGCCTTAAATGACTCT
*COL1A1*	TGGTCCCAAGGGTAACAGCG	AACACCAACAGGGCCAGGCT
*ALP*	ATGGGATGGGTGTCTCCACA	CCACGAAGGGGAACTTGTC
*GAPDH*	AAGGTCGGAGTCAACGGATTTG	TCCTGGAAGATGGTGATGGGAT

## Data Availability

All data are included in the manuscript. The datasets generated during and/or analyzed during the current study are available from the corresponding author on reasonable request.
